# Multi-Analyte Method for Antibiotic Residue Determination in Honey Under EU Regulation 2021/808

**DOI:** 10.3390/antibiotics14100987

**Published:** 2025-10-02

**Authors:** Helena Rodrigues, Marta Leite, Maria Beatriz P. P. Oliveira, Andreia Freitas

**Affiliations:** 1 Faculty of Pharmacy, University of Porto, Rua de Jorge Viterbo Ferreira 228, 4050-313 Porto, Portugal; up201805071@edu.ff.up.pt (H.R.); beatoliv@ff.up.pt (M.B.P.P.O.); 2 National Institute for Agricultural and Veterinary Research (INIAV), Rua dos Lagidos, Lugar da Madalena, 4485-655 Vila do Conde, Portugal; andreia.freitas@iniav.pt; 3 Associated Laboratory for Green Chemistry (LAQV) of the Network of Chemistry and Technology (REQUIMTE), Praça Coronel Pacheco nº15-6º, 4050-453 Porto, Portugal

**Keywords:** antibiotic, honey, multi-class approach, validation, QuEChERS, UHPLC-TOF-MS, commercial samples, analysis

## Abstract

**Background/Objectives:** Antibiotic detection in honey is challenging due to the complexity of this product, the typically low levels of residues, and the absence of Maximum Residue Levels (MRLs) for beehive products. The use of antibiotics in apiculture poses potential risks to human health, including antimicrobial resistance and toxic effects. Reliable, sensitive, and selective analytical methods are essential to ensure food safety and enable accurate monitoring of antibiotic contamination in honey. This study aimed to validate a multi-analyte procedure in accordance with the parameters established in Commission Implementing Regulation (EU) 2021/808 for the identification and quantification of antibiotics, including tetracyclines, lincosamides, quinolones, macrolides, β-lactams, sulfonamides, and diaminopyrimidines. **Methods**: An extraction protocol was developed using 0.1% formic acid in ACN:H_2_O (80:20, *v*/*v*), followed by a modified QuEChERS with the addition of 1 g NaCl and 2 g MgSO_4_. The extracts were analyzed by UHPLC-TOF-MS. **Results**: The method, validated under CIR (EU) 2021/808, demonstrated robust performance, with recoveries ranging from 80.1% to 117.6%, repeatability between 0.5% and 32.2%, reproducibility between 2.3% and 31.6%, and determination coefficients (R^2^) ranging from 0.9429 to 0.9982. Validation was achieved for 15 antibiotic residues, with CCβ from 3 to 15 μg·kg^−1^, LODs between 0.09 and 6.19 μg·kg^−1^, and LOQs between 0.29 and 18.77 μg·kg^−1^. Application to 10 commercial Portuguese honey revealed no detectable levels of the target antibiotics. **Conclusions**: The combination of a simplified extraction with UHPLC-TOF-MS provides a reliable approach for the determination of antibiotics in honey. This validated method represents a valuable tool for food safety monitoring and risk assessment of apiculture practices.

## 1. Introduction

Adding to their important role in the pollination of agricultural fields and maintaining plant biodiversity, bees—particularly honeybees—have other highly valued functions, namely producing beekeeping products such as honey, royal jelly, propolis, and beeswax, all of which have significant nutritional and commercial importance [1,2]. Among other environmental factors, namely pesticides, climate change, land use, bee management practices, and general pollution, several diseases affecting honeybees can lead to the loss of entire colonies, causing not only significant economic losses but also serious negative impacts on food production [3,4]. Like all living organisms, bees are vulnerable to a wide range of infectious agents, including viruses, fungi, or infesting parasitic mites, and, in the context of our study, bacteria [5]. Antibiotics may be used in beekeeping practices as a strategy to improve the overall health of the bee colony, to prevent the spread of disease, or to treat infections once they are identified, such as European and American foulbrood [6]. Compounds from tetracycline and sulfonamide families are among the antibiotics most mentioned in the literature, especially in countries where their application is allowed by regulation, unlike European countries, which restrict their use [7]. The application of veterinary drugs on bee hives can result in their presence in food of animal origin—for example, honey and other beehive products—representing risks to human and animal health, and to the environment [8]. The development and spread of antibiotic resistance, and the appearance of allergic reactions, severe conditions like organ toxicity, and reproductive disorders, are some of the described problems directly associated with consuming food containing antibiotic residues [9].

In line with the importance of ensuring food safety and promoting safer consumption practices, the European Commission has established Maximum Residue Limits (MRLs) for pharmacologically active substances, including anti-infectious agents/antibiotics, in foodstuffs of animal origin. These limits apply to target tissues such as liver, muscle, milk, kidney, fat, and eggs, but not to honey or other beehive products [10]. The accurate detection of antibiotic residues relies on these validated and reliable methods that comply with regulations concerning the maximum permitted levels of the target compounds and validation procedures [11]. Several analytical challenges can be identified in the development of methods for detecting antibiotic residues in foods, particularly in honey. Honey is a highly viscous and complex matrix, containing mainly sugars, along with smaller amounts of proteins, organic acids, and enzymes [12]. The composition of honey may interfere with both extraction and detection method efficiency, as some components may co-elute with the target antibiotics or enhance or suppress the signal, potentially compromising the accuracy of analyte identification and quantification [13]. Typically, antibiotic residues are present in honey and other food of animal origin at trace levels at concentrations of μg·kg^−1^ or lower [14]. Another difficulty is the fact that there are no established MRLs for antibiotic residues in beehive products, making it challenging to develop studies that comply with the final goal of conducting risk assessments because of the unclear regulatory reference points.

Given the regulatory context and analytical challenges, effective control of these anthropogenic contaminants requires the optimization and validation of analytical methods that are reliable, precise, accurate, and time efficient.

Regarding the methodological context and according to the literature, some of the most applied analytical procedures for screening veterinary drugs in honey and other food products are immunochemical assays—more specifically, ELISA (Enzyme-Linked ImmunoSorbent Assay) [15,16]—and microbiological methods such as inhibition-based tests [17]. Nonetheless, confirmatory methods enable unequivocal and accurate identification of compounds, and other mentioned specifications and the principal choices are Ultra-High-Performance Liquid Chromatography coupled with Mass Spectrometry (UHPLC-MS) [18,19]. More recently, High-Resolution Mass Spectrometry (HRMS) has demonstrated the required specificity and sensitivity for analysis of both targeted and untargeted analyses in a multi-detection approach, permitting the detection of a broad spectrum of antibiotic residues [20,21,22]. The complexity of the composition of food products, including honey, and the presence of minute quantities of antibiotic residues therein, render it imperative to implement an initial treatment of the sample to extract the analytes. Subsequently, purification or clean-up procedures are typically employed to obtain extracts with the minimum number of interferents, thereby facilitating more accurate detection and quantification data through the utilization of the analytical techniques employed [23]. Nowadays, multi-residue methods are increasingly used in routine analysis, as this approach allows for the simultaneous determination of several target analytes in a single run [24].

This study aimed to validate a previously optimized multi-class semi-quantitative screening analytical methodology for the identification of seven classes of antibiotic residues, which included macrolides, β-lactams, quinolones, sulfonamides, lincosamides, and tetracyclines in honey samples, specifically cefacetrile, cefoperazone, danofloxacin, cinoxacin, enrofloxacin, lomefloxacin, ciprofloxacin, trimethoprim, tylosin A, sulfadimethoxine, sulfadimidine, sulfathiazole, lincomycin, tetracycline, and oxytetracycline. The determination of LOD and LOQ values was also carried out during the validation of the applied method, consisting of the analysis of the honey samples previously extracted by a QuEChERS extraction procedure followed by analysis in a UHPLC-TOF-MS system. The contamination levels in real honey samples available to consumers were evaluated using the fully validated method described in the following sections through a small survey conducted by analyzing commercially available samples from several Portuguese supermarkets.

## 2. Results and Discussion

### 2.1. Compound Identification

According to Commission Implementing Regulation (EU) 2021/808 of 22 March 2021, the identification of antibiotics using UHPLC-ToF-MS must comply with the two criteria [25]. A review of the data in Table 1 reveals that 15 of the targeted analytes in the spiked representative samples were successfully identified, with no interference from unwanted compounds. These identifications met the specified criteria of an exact mass deviation of within 5 ppm and an RRT maximum deviation of below 1.0%. The maximum observed deviations were 4.17 ppm for sulfadimidine and 0.46% for cefacetrile. These results demonstrate that the specified criteria were met regarding both the exact mass deviation and the RRT maximum deviation.

### 2.2. Validation Parameters

The specificity and selectivity of the methodology were ascertained, thus enabling the identification and quantification of the antibiotics under investigation with good accuracy. The analysis of the blank samples (S/N > 3) revealed the absence of any interfering peaks considering a ±0.5 min window of the RT of each target compound, while the spiked blank samples revealed successful identification.

Table 2 displays the results found for the method validation study for the 15 antibiotics, including retention times, tested concentration ranges, calibration curve linearity (expressed as R^2^ values), concentration levels of the spiked blank samples analyzed in triplicate, precision data (repeatability and reproducibility), recoveries rates, CCβ values, and the determined LOD and LOQ.

An analysis of the validation results in Table 2 shows that the calibration curve linearity, expressed as R^2^ values, ranged from 0.9429 to 0.9982. In terms of precision values represented by repeatability, it was found that the lowest value, corresponding to 0.5%, was obtained for cinoxacin, while the highest value, corresponding to 32.3%, was obtained for ciprofloxacin. The variation between the minimum and maximum precision values can be explained by the differential matrix effects of honey on extraction efficiency and ionization across analytes; consequently, some compounds, such as ciprofloxacin, exhibited considerably higher variability than others. In addition, the precision of the validation procedure was evaluated by repeating the procedure over three days, thus assessing the reproducibility. The lowest value was observed for sulfadimethoxine (2.3%), while ciprofloxacin presented the highest value (31.6%). Overall, the antibiotics showed satisfactory repeatability and reproducibility values, with an average of 13.2% and 13.8%, respectively. Recovery values obtained were generally satisfactory, with an average of 101.7%, and for all 15 antibiotics, they were within the acceptable range, between 80 and 120%. The lowest recovery was observed for tetracycline (80.1%), while enrofloxacin showed the highest (117.6%). CCβ assessed values ranged from 3 to 15 μg·kg^−1^ for the 15 compounds. An analysis of the LOD and LOQ data showed that lomefloxacin had the lowest values, at 0.09 μg·kg^−1^ and 0.29 μg·kg^−1^, respectively, while cinoxacin exhibited the highest values, at 6.19 μg·kg^−1^ and 18.77 μg·kg^−1^.

The determination of antibiotics in honey by applying modified QuEChERS protocols has been reported by several authors with slight modifications regarding the extraction conditions, namely the type of sorbents and salts used as well as extraction solvents, normally followed by separation and detection of the target compounds using analytical techniques such as UHPLC-MS/MS. According to these authors, validation studies were conducted, the parameters were acquired for antibiotics belonging to the families studied here, and the resulting data for the regulatory parameters defined were generally consistent with those obtained in our validation work.

Varenina et al. [26] successfully extracted antibiotics from the quinolone, macrolide, tetracycline, and sulfonamide families, reporting minimum recoveries of 94.2% and maximum recoveries of 104.1%. The method demonstrated a repeatability of below 30%, and satisfactory method linearity was achieved, with R^2^ ranging from 0.96 to 9.98, in agreement with the results attained in the present study. Xu et al. [27] validated an extraction QuEChERS method, with LOD values ranging from 0.24 to 0.68 μg·kg^−1^ and LOQ values from 0.80 to 2.27 μg·kg^−1^ for antibiotics belonging to the macrolide and lincosamide families, which are comparable to those observed in this study. The method demonstrated excellent recoveries, with values ranging from 81.3% to 99.0%, high linearity (R^2^ values between 0.9991 and 1.000), and relative standard deviations (RSDs) below 9.18%. Yang et al. [28] applied and validated a multiclass method for analyzing antibiotics from macrolide, beta-lactam, quinolone, tetracycline, and sulfonamide families. The method showed intra-day RSD values lower than 10.0%, inter-day RSD values lower than 13.9%, and recoveries with a minimum value of 70.5%, lower than what we obtained, and a maximum value of 119.8%, more approximate to our results. The reported LOD and LOQ values ranged from 0.050 to 1.02 μg·kg^−1^ and from 0.17 to 3.40 μg·kg^−1^, respectively.

In addition to the use of multiclass approaches, several studies have specifically evaluated the efficiency of extraction methods designed for individual classes of antibiotics. As an example, Lei et al. [29] validated a method for quinolone determination, with recoveries between 81.8% and 116.8%, LOD values ranging from 0.77 to 0.99 μg·kg^−1^, and LOQ values ranging from 3.07 to 4.13 μg·kg^−1^. Furthermore, the method exhibited excellent linearity, with correlation coefficients (R^2^) exceeding 0.990.

### 2.3. Analysis of Commercial Portuguese Samples

The applicability of the validated analytical method was assessed by applying the same extraction protocol and analytical conditions, as described in the Method Validation section, to ten commercial honey samples. The ten samples were evaluated to study the presence of antibiotic residues, using the validated multiclass approach. Overall, none of the target antibiotic residues were identified in the samples at levels above the method’s Limit of Detection.

Although the target analytes were not detected in the samples analyzed, several published studies have reported the presence of this type of compound in commercially available honey.

Gün et al. [30] analyzed 22 honey samples purchased from Turkish supermarkets for the presence of 29 antibiotic residues, including compounds from the tetracycline, aminoglycoside, macrolide, sulfonamide, fluoroquinolone, benzimidazole, anthelmintic, amphenicol, quinoline, and oxazolidine groups. The quantification of the target analytes was carried out using an LC-QTRAP-MS/MS system, and the results demonstrated that 10 out of 22 honey samples were positive for at least one of the antibiotic residues. Dihydrostreptomycin was detected in six samples, with concentrations in the range of 9.94 to 992.58 μg·kg^−1^; erythromycin in three samples, at concentrations between 4.12 and 19.16 μg·kg^−1^; streptomycin in two samples, at 7.50 and 7.75 μg·kg^−1^; sulfadimidine in six samples, at concentrations from 2.16 to 196.51 μg·kg^−1^; and tetracycline in one sample, at a concentration of 20.44 μg·kg^−1^.

A “two-procedure approach” using LC-MS/MS detection was employed by Paoletti et al. [31] to evaluate the occurrence of 64 antibiotic residues from eight classes, namely lincosamides, amphenicols, macrolides, pleuromutilins, quinolones, nitroimidazoles, tetracyclines, and sulfonamides, in honey, mostly of European origin as well as in samples of unknown origin or blends of honey samples from multiple countries. Of the 55 honey samples purchased from local markets, three were positive for at least one antibiotic residue: a mixed honey from Moldova, Argentina, Taiwan Romania, and Ukraine tested positive for tetracycline and sulfamethazine at concentrations of 0.5 and 1.3 μg·kg^−1^, one Italian sample contained oxytetracycline and tetracycline at concentrations of 1.1 and 0.5 μg·kg^−1^, and a third sample, a mixed honey from Ukraine and Hungary, was found to contain 0.5 μg·kg^−1^ of sulfathiazole.

Wang et al. [32] described an analytical approach for the confirmation and quantification of 40 veterinary drugs from four classes in honey, using QuEChERS as the extraction method, followed by compound analysis with an LC-Q-TOF/MS system. Twelve commercial samples, randomly acquired from a local store, were screened for the target compounds, and one honey sample was found to be positive for the presence of ciprofloxacin, with a determined concentration of 99.7 μg·kg^−1^.

A study conducted in Brazil by Perin et al. [33] analyzed 96 honey samples collected from different regional locations of the country (Northeast, Central–West, Southeast, and South) for the occurrence of 14 antibiotic residues, including aminoglycosides, amphenicols, fluoroquinolones, macrolides, sulfonamides, and tetracyclines. The results showed that only one sample contained an antibiotic residue—enrofloxacin, belonging to the fluoroquinolone antibiotic family—at a concentration below the method’s LOQ (<5 μg·kg^−1^).

## 3. Materials and Methods

### 3.1. Chemicals and Reagents

Ultrapure water was produced by a Millipore System (France). Acetonitrile (ACN) was purchased from PanReac AppliChem ITW Reagents (Monza, Italy). Sodium chloride (NaCl) and magnesium sulphate (MgSO_4_) were obtained from Honeywell Fluka (Dusseldorf, Germany), and formic acid (with a purity between 99 to 100%) was acquired from Chem-Lab (Zedelgem, Belgium). Antibiotic standards from 7 distinct groups comprised trimethoprim (diaminopyrimidines); cefoperazone and cefacetrile (cephalosporins); tylosin A (macrolides), sulfadimidine, sulfadimethoxine, and sulfathiazole (sulfonamides); lincomycin (lincosamide); oxytetracycline and tetracycline (tetracyclines); and ciprofloxacin, cinoxacin, enrofloxacin, danofloxacin, and lomefloxacin (fluoroquinolones). Internal standards for each class in the analysis were purchased from Sigma-Aldrich (Madrid, Spain) and included rufloxacin, cefadroxil-d_4_, penicillin G-d_7_, erythromycin-^13^C-d_3_, sulfadiazine-^13^C_6_, minocycline, and trimethoprim-d_9_) (purity > 98%).

### 3.2. Instrumentation

Besides the glassware material typically used in laboratory activities, the equipment included 50 mL and 15 mL centrifuge tubes and micropipettes of variable volumes. The sample preparation procedure required several pieces of equipment, including balances from Mettler Toledo AE100 and PC200 (Greifensee, Switzerland), a ZX3 vortex mixer from Velp Scientifica (Usmate, Italy), a Heidolph REAX 2 rotary mixer (Schwabach, Germany), a refrigerated centrifuge from the brand Heraeus Megafuge 1.0 (Hanau, Germany), an Turbovap Zymark evaporator with a nitrogen generator (Hopkinton, MA, USA), and a Bandelin Sonorex Super RK510 ultrasonic bath (Berlin, Germany).

For method validation, an Ultra-High-Resolution Liquid Chromatography coupled with Mass Spectrometry with a Time-of-Flight analyzer (UHPLC-ToF-MS) was used as reported in Rodrigues et al. (2025) [34]. Analyte separation was accomplished by using a reverse–phase Acquity UPLC HSS T3 column (1.8 µm, 2.1 × 100 mm) acquired from Waters (Milford, MA, USA) integrated into a Nexera X2 UHPLC system (Shimadzu, Japan). The chromatographic system encompassed a binary pump, a temperature-controlled autosampler, a solvent degasser, a column oven, and an automatic injector with a variable volume. Chromatographic conditions were defined as follows: column temperature at 40 °C, autosampler temperature of 10 °C, flow rate of 500 µL.min^−1^, and sample injection volume of 10 µL. The mobile phase constituted 0.1% formic acid in water (solvent A) and acetonitrile (solvent B), and the gradient elution program was as follows: 97% (A) (0–2 min), 97 to 40% (A) (5 min), 40 to 0% (A) (9 min), and 0 to 97% (A) (10–11 min). Analyte detection by mass spectrometry was performed by a Triple TOF^TM^ 5600 + MS from the Sciex brand (Framingham, MA, USA) in positive ionization mode (ESI^+^). Data were acquired in full-scan acquisition mode in a mass range of 100–900 Da. Detection settings included a curtain gas (CUR) pressure of 35 psi, gas 1 and 2 pressures at 40 psi, a source temperature of 550 °C, and an IonSpray voltage of 5500 V. The software Analyst^®^ TF 1.7 software for data acquisition, PeakView™ 2.2, and LibraryView™ 1.3 software, for analyte identification, and MultiQuant™ 3.0 for quantification, were provided by Sciex.

For analyte identification, two parameters were assessed: the exact mass deviation (Δppm) of the precursor ion and the maximum deviation of the relative retention time (ΔRRT), in accordance with the performance criteria set in Commission Implementing Regulation (EU) 2021/808 [10] and calculated using the following equations:

(1) $$∆ppm=\left(\frac{Mass\;detected-Exact\;mass}{Exact\;mass}\right)\;\times \;10^{6},$$

(2) $$∆RRT\left(\%\right)=\left(\frac{{RRT}_{sample}-{RRT}_{standard}}{{RRT}_{standard}}\right)\times 100,$$

### 3.3. Standard Solutions

Formic acid 0.1% solution was prepared by adding an appropriate volume of concentrated formic acid (99–100%) to an ACN:H_2_O mixture (80:20, *v*/*v*), which had been previously prepared by adding specific volumes of acetonitrile and water. Formic acid at 0.1% was prepared by dilution of 1 mL of concentrated formic acid (99–100%) in ultra-pure water to a final volume of 1 L. The resulting solution was filtered using an HPLC eluent filtration system equipped with a PVDF membrane (0.45 µm, 13 mm) under vacuum, followed by degassing with ultrasound for 15 min. Mobile phase B (acetonitrile) was subjected to the same filtration and degassing procedures.

The validation process included the preparation of standard solutions for spiking purposes, including mixed solutions containing two or more families of antibiotics and working solutions prepared from these. Working solutions were prepared considering the validation level set for each of the 15 antibiotic residues in the study.

The internal standard solution (IS) was prepared in MeOH using previously prepared stock solutions of penicillin G-d_7_, cefadroxil-d_4_, and erythromycin-^13^C-d_3_ at a concentration of 20 μg·mL^−1^ and minocycline, sulfadiazine-^13^C_6_, rufloxacin, and trimethoprim-d_9_ at a concentration of 10 μg·mL^−1^. All standard solutions were stored at −30 °C until further use.

### 3.4. Sample Preparation and Extraction Protocol

The honey was acquired in supermarket stores in Portugal and stored at −20 ± 2 °C until analysis. The protocol used followed our previous work [34]. Briefly, honey samples (2.0 ± 0.05 g) were weighed into 50 mL centrifuge tubes and a volume of 50 μL of IS solution was added, including the 20 blank and spiked blank samples. The extraction procedure began with the addition of 10 mL of 0.1% formic acid in ACN:H_2_O (80:20, *v*/*v*), with further homogenization for 15 s in vortex and 20 min in a reax shaker. Afterwards, 1 g of NaCl and 2 g of MgSO_4_ were added to the solid–liquid mixtures as part of the salting-out phase. After 1 min of vortex, the tubes were submitted to centrifugation at 4500 rpm for 10 min at 4 °C. Five mL of the supernatant was transferred to a clean tube and then evaporated under a nitrogen stream at 40 °C until a final volume of 500 μL was reached. Subsequently, 200 μL of 0.1% formic acid was added, followed by vortex agitation for 15 s. A final filtration step was carried out by passing the solution through a PVDF Mini-Uniprep TM filter (0.45 μm), with further injection into the UHPLC-ToF-MS system.

### 3.5. Method Validation

In the absence of established Maximum Residue Limits (MRLs) for the antibiotics under investigation in honey or other apiculture products, as specified in Regulation 37/2010 for other foodstuffs of animal origin (e.g., eggs, muscle, milk), the lowest level defined for each antibiotic was designated as the validation level (VL) for honey. In general, the lowest MRLs are set for target matrices such as milk (cefacetrile—125 μg·kg^−1^; danofloxacin—30 μg·kg^−1^; cefoperazone, ciprofloxacin, enrofloxacin, tylosin A, and trimethoprim—50 μg·kg^−1^; and sulfadimethoxine, sulfadimidine, and sulfathiazole—100 μg·kg^−1^) or eggs (lincomycin—50 μg·kg^−1^).

In cases where no MRL had been established for any matrix, the lowest level applicable within the same antibiotic family was selected. This criterion was applied to cinoxacin and lomefloxacin, for which a value of 30 μg·kg^−1^ was selected [10]. This approach followed the cascade MRLs defined in Commission Implementing Regulation (EU) 2018/470 [35]. Before carrying out the method validation procedures, for quality control purposes a set of samples was evaluated to check for blank matrices. These samples were then used for spiking procedures.

Commission Implementing Regulation (EU) 2021/808 specifies the parameters required for the validation of semi-quantitative screening methods, including the selectivity/specificity, precision (repeatability and reproducibility), recovery (or trueness), calibration curves ranging from 0.1 VL to 2 VL (linearity), and CCβ values. Although not included in the mentioned regulation, analytical limits of the equipment for detection, namely the Limit of Detection (LOD), and for quantification, which namely the Limit of Quantification (LOQ), were also determined.

The specificity and selectivity of the proposed analytical method were evaluated by examining a representative set of blank samples (n = 20) and searching for any interfering peaks/signals in the retention time (RT) of the target analyte. A matrix calibration curve with five concentration levels was prepared by the addition of specific volumes of the working solution to blank honey aliquots prior to extraction (0.1 × VL, 0.5 × VL, 1 × VL, 1.5 × VL and 2 × VL). The data resulting from the three-day analysis of the spiked blank samples used for calibration curve construction were employed to calculate recoveries and precision values.

Recovery can be defined as the “true” analyte concentration that is recovered during an analytical procedure and that is present in the final sample extract, and it is calculated by dividing the measured level (the analyte concentration calculated directly from the calibration curve) by the spiked level (the theoretical analyte concentration after spiking the blanks) in percentage.

Levels 2, 3, and 4 were performed in triplicate to determine the precision parameters expressed in terms of the coefficient of variation (CV), Intra CV (%) for repeatability, and Inter CV (%) for reproducibility, in percentage, and calculated by dividing the standard deviation by the mean of the results.

Detection capacity (CCβ) is defined as “the lowest analyte content that can be detected or quantified in a sample with a probability of error β.” The beta error (β) corresponds to “the probability that the analyzed sample is in fact non-compliant, although a compliant measurement result has been obtained,” and must be equal to or less than 5%. In this study, CCβ was the concentration capable of guaranteeing a false negative rate of ≤5%. Accordingly, it was defined as the lowest point of the calibration curve and verified to comply with this criterion.

The Limit of Detection (LOD) can be defined as the minimum measured level at which the presence of the compound can be detected with a suitable statistical confidence, while the Limit of Quantification (LOQ) is the lowest measured concentration at which the compound can be quantitatively determined with the required precision and accuracy. The LOD and LOQ were evaluated according to the following equations, where SD_Blank samples_ represents the standard deviation value of the peak area of the 20 analyzed blank samples, and m is the slope of the calibration curve.

(3) $$LOD=3.3\times \frac{SD\;Blank\;samples}{m},$$

(4) $$LOQ=10\times \frac{SD\;Blank\;samples}{m}$$

## 4. Conclusions

In the validation study, the method determined to be the most effective, following optimization under multiple hypotheses, was selected for application. This entailed the use of an extraction step using formic acid 0.1% prepared in ACN:H_2_O (80:20, *v*/*v*) as the extraction solvent, followed by the application of a QuEChERS procedure to the resulting extract, whereby MgSO_4_ and NaCl were added without incorporating a dispersive-SPE step.

The semi-quantitative screening method was found to be an appropriate means of monitoring 15 antibiotic residues in laboratory settings. These included members of the tetracyclines (tetracycline and oxytetracycline), diaminopyrimidines (trimethoprim), cephalosporins (cefoperazone and cefacetrile), macrolides and lincosamides (tylosin A and lincomycin), quinolones (cinoxacin, danofloxacin, lomefloxacin ciprofloxacin, and enrofloxacin), and sulfonamides (sulfadimidine, sulfadimethoxine, sulfathiazole) in food products such as honey.

About the 15 antibiotics under study, it can be stated that the performance criteria established in Commission Implementing Regulation (EU) 2021/808 of 22 March 2021 have been met, which include the linearity of the calibration curve obtained, the precision criteria (repeatability and reproducibility), recovery, CCβ values, and analytical limits of the method (LOD and LOQ). Although none of the ten retail honey samples contained detectable antibiotic residues, the limited sample size (n = 10) restricted inference; therefore, a larger, regionally and seasonally stratified survey will be undertaken to obtain a more representative picture of contamination levels. In this context, there is a continuous need to guarantee the safety of this widely consumed food product, which is of utmost importance in a globalized scenario where regulations on antibiotic use vary geographically. To maintain accurate control and monitoring of such samples, validation of the analytical methodologies is crucial to proceeding with appropriate management strategies and the development of appropriate mitigation plans.

## Figures and Tables

**Table 1:** Identification parameters of the 15 compounds under study by UHPLC-ToF-MS.

Compound	Formula	Exact Mass (Da)	RT (min)	RRT Maximum Deviation (%)	Exact Mass Deviation (ppm)
Cefacetrile	C_13_H_13_N_3_O_6_S	340.0599	4.46	0.46	0.48
Cefoperazone	C_25_H_27_N_9_O_8_S_2_	646.1490	4.46	0.32	−0.98
Trimethoprim	C_14_H_18_N_4_O_3_	291.1452	3.97	0.04	−0.02
Cinoxacin	C_12_H_10_N_2_O_5_	263.0639	4.65	0.12	−4.80
Ciprofloxacin	C_17_H_18_FN_3_O_3_	332.1408	4.04	0.06	0.91
Danofloxacin	C_19_H_20_FN_3_O_3_	358.1563	4.09	0.01	0.56
Enrofloxacin	C_19_H_22_FN_3_O_3_	360.1721	4.14	0.01	0.75
Lomefloxacin	C_17_H_19_F_2_N_3_O_3_	352.1466	4.10	0.00	−0.40
Tylosin A	C_46_H_77_NO_17_	916.5248	4.91	0.03	−1.81
Lincomycin	C_18_H_34_N_2_O_6_S	407.2223	3.79	0.00	3.14
Sulfadimethoxine	C_12_H_14_N_4_O_4_S	311.0806	4.92	0.04	−0.91
Sulfadimidine	C_15_H_15_N_5_O_2_S_2_	279.0925	5.63	0.01	4.17
Sulfathiazole	C_9_H_9_N_3_O_2_S_2_	256.0209	3.87	0.27	−0.02
Oxytetracycline	C_22_H_24_N_2_O_9_	461.1552	4.04	0.10	−0.51
Tetracycline	C_22_H_24_N_2_O_8_	445.1604	4.14	0.04	−0.36

**Table 2:** Performance criteria for method validation of 15 antibiotic residues in honey.

Compound	RT (min.)	Range (μg·kg^−1^)	Linearity (R^2^ Value)	Levels (μg·kg^−1^) (n = 6)	Repeatability (%)	Reproducibility (%)	Recovery (%)	CCβ (μg·kg^−1^)	LOD (μg·kg^−1^)	LOQ (μg·kg^−1^)
Cefacetrile	4.00	12.5–250	0.9891	62.5	16.6	27.4	86.5	12.5	1.52	4.59
				125	4.6	5.6	99.0			
				187.5	6.2	7.6	103.5			
Cefoperazone	4.46	5–100	0.9898	25	9.6	24.2	98.2	5	1.10	3.35
				50	1.9	8.3	95.1			
				75	13.1	12.5	105.2			
Cinoxacin	4.66	15–60	0.9801	15	11.1	10.9	101.1	15	6.19	18.77
				30	0.5	2.5	111.8			
				45	11.4	9.8	106.2			
Ciprofloxacin	4.03	5–100	0.9905	25	32.3	31.6	89.9	5	0.41	1.23
				50	13.1	10.8	112.1			
				75	22.9	20.0	101.3			
Danofloxacin	4.09	3–60	0.9662	15	1.6	13.3	95.2	3	0.29	0.87
				30	9.8	9.3	117.5			
				45	9.7	10.8	107.6			
Enrofloxacin	4.14	5–100	0.9429	25	21.4	18.6	90.7	5	0.14	0.43
				50	7.2	6.7	109.5			
				75	9.5	9.5	117.6			
Lomefloxacin	4.09	3–60	0.9783	15	12.3	14.8	92.5	3	0.09	0.29
				30	11.5	10.0	104.9			
				45	11.5	12.7	111.0			
Trimethoprim	3.96	5–100	0.9957	25	23.4	27.1	90.5	5	0.20	0.62
				50	9.1	8.1	102.3			
				75	14.6	15.5	104.5			
Tylosin A	4.91	5–100	0.9702	25	27.9	24.4	101.4	5	0.14	0.42
				50	7.0	7.7	108.8			
				75	18.3	18.4	111.0			
Lincomycin	3.79	5–100	0.9849	25	4.4	4.7	108.1	5	0.64	1.93
				50	8.2	9.8	104.7			
				75	18.6	22.6	106.2			
Sulfadimethoxine	4.92	10–200	0.9915	50	10.2	11.5	95.7	10	0.10	0.29
				100	2.9	2.3	96.3			
				150	11.2	12.9	107.8			
Sulfadimidine	4.26	10–200	0.9647	50	24.4	14.9	85.2	10	1.30	3.95
				100	11.6	10.5	103.0			
				150	19.3	14.4	115.0			
Sulfathiazole	3.87	10–200	0.9913	50	17.9	21.1	91.2	10	0.17	0.52
				100	6.3	4.7	104.6			
				150	16.1	15.3	106.4			
Oxytetracycline	4.03	10–200	0.9982	50	27.8	14.6	100.0	10	0.96	2.91
				100	20.0	17.5	97.9			
				150	4.5	13.7	103.6			
Tetracycline	4.13	10–200	0.9964	50	17.2	20.1	80.1	10	2.13	6.47
				100	16.3	13.1	96.5			
				150	19.7	19.2	101.1			

## Data Availability

The original contributions presented in this study are included in the article. Further inquiries can be directed at the corresponding author(s).

## References

[1] Baffoni L., Alberoni D., Gaggìa F., Braglia C., Stanton C., Ross P.R., Di Gioia D. (2021). Honeybee Exposure to Veterinary Drugs: How Is the Gut Microbiota Affected?. Microbiol. Spectr..

[2] Kumar R., Kumari S., Saxena A. (2024). Role of Honeybees in Improving Biodiversity and Sustainable Source of Income. J. Entomol. Res..

[3] Hristov P., Shumkova R., Palova N., Neov B. (2020). Factors Associated with Honey Bee Colony Losses: A Mini-Review. Vet. Sci..

[4] Margaoan R., Papa G., Nicolescu A., Cornea-Cipcigan M., Kösoğlu M., Topal E., Negri I. (2024). Environmental Pollution Effect on Honey Bees and Their Derived Products: A Comprehensive Analysis. Environ. Sci. Pollut. Res..

[5] Ullah A., Gajger I.T., Majoros A., Dar S.A., Khan S., Shah A.H., Khabir M.N., Hussain R., Khan H.U., Hameed M. (2021). Viral Impacts on Honey Bee Populations: A Review. Saudi J. Biol. Sci..

[6] Ortiz-Alvarado Y., Clark D.R., Vega-Melendez C.J., Flores-Cruz Z., Domingez-Bello M.G., Giray T. (2020). Antibiotics in Hives and Their Effects on Honey Bee Physiology and Behavioral Development. Biol. Open.

[7] Reybroeck W., Daeseleire E., De Brabander H.F., Herman L. (2012). Antimicrobials in Beekeeping. Vet. Microbiol..

[8] Arsène M.M.J., Davares A.K.L., Viktorovna P.I., Andreevna S.L., Sarra S., Khelifi I., Sergueïevna D.M. (2022). The Public Health Issue of Antibiotic Residues in Food and Feed: Causes, Consequences, and Potential Solutions. Vet. World.

[9] Bacanlı M.G. (2024). The Two Faces of Antibiotics: An Overview of the Effects of Antibiotic Residues in Foodstuffs. Arch. Toxicol..

[10] European Commission (2010). Commission Regulation (EU) No 37/2010 of 22 December 2009 on Pharmacologically Active Substances and Their Classification Regarding Maximum Residue Limits in Foodstuffs of Animal Origin. Off. J. Eur. Communities.

[11] Pratiwi R., Ramadhanti S.P., Amatulloh A., Megantara S., Subra L. (2023). Recent Advances in the Determination of Veterinary Drug Residues in Food. Foods.

[12] Bargańska Ż., Namieśnik J., Ślebioda M. (2011). Determination of Antibiotic Residues in Honey. TrAC Trends Anal. Chem..

[13] Kujawski M.W., Namieśnik J. (2008). Challenges in Preparing Honey Samples for Chromatographic Determination of Contaminants and Trace Residues. TrAC Trends Anal. Chem..

[14] Ghimpețeanu O.M., Pogurschi E.N., Popa D.C., Dragomir N., Drăgotoiu T., Mihai O.D., Petcu C.D. (2022). Antibiotic Use in Livestock and Residues in Food—A Public Health Threat: A Review. Foods.

[15] Jakšić S., Mihaljev Ž., Kartalović B., Babić J., Vidaković S., Baloš-Živkov M. (2018). Evaluation of ELISA Tests as Screening Methods for Determination of Antibiotics and Sulfonamides in Honey. Food Feed. Res..

[16] Mahmoudi R., Norian R., Pajohi-Alamoti M. (2014). Antibiotic Residues in Iranian Honey by Elisa. Int. J. Food Prop..

[17] Wu Q., Shabbir M.A.B., Peng D., Yuan Z., Wang Y. (2021). Microbiological Inhibition-Based Method for Screening and Identifying of Antibiotic Residues in Milk, Chicken Egg and Honey. Food Chem..

[18] Orso D., Floriano L., Ribeiro L.C., Bandeira N.M.G., Prestes O.D., Zanella R. (2016). Simultaneous Determination of Multiclass Pesticides and Antibiotics in Honey Samples Based on Ultra-High Performance Liquid Chromatography-Tandem Mass Spectrometry. Food Anal. Methods.

[19] Schwaiger B., König J., Lesueur C. (2018). Development and Validation of a Multi-Class UHPLC-MS/MS Method for Determination of Antibiotic Residues in Dairy Products. Food Anal. Methods.

[20] Igualada C., Giraldo J., Font G., Yusà V. (2022). Validation of a Multi-Residue UHPLC-HRMS Method for Antibiotics Screening in Milk, Fresh Cheese, and Whey. J. Food Compos. Anal..

[21] Leite M., Marques A.R., Pouca A.S.V., Barros S.C., Barbosa J., Ramos F., Afonso I.M., Freitas A. (2023). UHPLC-ToF-MS as a High-Resolution Mass Spectrometry Tool for Veterinary Drug Quantification in Milk. Separations.

[22] Spörri A.S., Jan P., Cognard E., Ortelli D., Edder P. (2014). Comprehensive Screening of Veterinary Drugs in Honey by Ultra-High-Performance Liquid Chromatography Coupled to Mass Spectrometry. Food Addit. Contam. Part A.

[23] Chen Z., Daka Z., Yao L., Dong J., Zhang Y., Li P., Zhang K., Ji S. (2025). Recent Progress in the Application of Chromatography-Coupled Mass-Spectrometry in the Analysis of Contaminants in Food Products. Food Chem. X.

[24] Shin D., Kim J., Kang H.-S. (2021). Simultaneous Determination of Multi-Pesticide Residues in Fish and Shrimp Using Dispersive-Solid Phase Extraction with Liquid Chromatography–Tandem Mass Spectrometry. Food Control.

[25] European Commission (2021). Commission Implementing Regulation (EU) 2021/808 of 22 March 2021 on the Performance of Analytical Methods for Residues of Pharmacologically Active Substances Used in Food-Producing Animals and on the Interpretation of Results as Well as on the Methods to Be Used for Sampling and Repealing Decisions 2002/657/EC and 98/179/EC. Off. J. Eur. Union.

[26] Varenina I., Bilandžić N., Luburić Đ.B., Kolanović B.S., Varga I., Sedak M., Đokić M. (2023). Determination of Quinolones, Macrolides, Sulfonamides and Tetracyclines in Honey Using QuEChERS Sample Preparation and UHPLC-MS/MS Analysis. Food Control.

[27] Xu J., Yang M., Wang Y., Yang Y., Tu F., Yi J., Hou J., Lu H., Jiang X., Chen D. (2021). Multiresidue Analysis of 15 Antibiotics in Honey Using Modified QuEChERS and High Performance Liquid Chromatography-Tandem Mass Spectrometry. J. Food Compos. Anal..

[28] Yang Y., Lin G., Liu L., Lin T. (2022). Rapid Determination of Multi-Antibiotic Residues in Honey Based on Modified QuEChERS Method Coupled with UPLC–MS/MS. Food Chem..

[29] Lei H., Guo J., Lv Z., Zhu X., Xue X., Wu L., Cao W. (2018). Simultaneous Determination of Nitroimidazoles and Quinolones in Honey by Modified QuEChERS and LC-MS/MS Analysis. Int. J. Anal. Chem..

[30] Gün R., Dursun İ., Arıcı B., Saraç Y. (2024). Detection of Multiple Antibiotic Residues in Turkish Pine and Blossom Honeys Using LC–MS/MS Method. Chem. Biodivers.

[31] Paoletti F., Sdogati S., Barola C., Giusepponi D., Moretti S., Galarini R. (2022). Two-Procedure Approach for Multiclass Determination of 64 Antibiotics in Honey Using Liquid Chromatography Coupled to Time-of-Flight Mass Spectrometry. Food Control.

[32] Wang Z., Li Y., Chang Q., Kang J., Pang G.-F. (2018). A Detection and Confirmation Strategy for Screening of Veterinary Drugs in Honey by Liquid Chromatography Coupled Quadrupole Time-of-Flight Mass Spectrometry. Anal. Methods.

[33] Perin M., Barnet L.S., da Costa J.S., Barreto F. (2023). Determination of Different Antimicrobial Classes Residues in Honey Using a Simple LLE Technique and Clean-Up Dispersive SPE Couple LC–MS/MS: Application in Sample of Different Regions from Brazil. Food Anal. Methods.

[34] Rodrigues H., Leite M., Oliveira B., Freitas A. (2025). Development and Optimization of Extraction Methodologies for the Determination of Antibiotics in Honey by UHPLC-ToF-MS. J. AOAC Int..

[35] European Commission (2018). Commission Implementing Regulation (EU) 2018/470 of 21 March 2018 on Detailed Rules on the Maximum Residue Limit to Be Considered for Control Purposes for Foodstuffs Derived from Animals Which Have Been Treated in the EU under Article 11 of Directive 2001/82/EC. Off. J. Eur. Union.

